# Perceived Social Support and Sustained Physical Activity During the COVID-19 Pandemic

**DOI:** 10.1007/s12529-022-10125-2

**Published:** 2022-09-29

**Authors:** Verity Hailey, Abi Fisher, Mark Hamer, Daisy Fancourt

**Affiliations:** 1 Department of Behavioural Science and Health, University College London, 1-19 Torrington Place, London, WC1E 7HB UK; 2 Institute Sport Exercise & Health, Division Surgery & Interventional Science, University College London, London, UK

**Keywords:** Social support, Loneliness, Social isolation, Physical activity, Lockdown

## Abstract

**Background:**

COVID-19 lockdown introduced substantial barriers to physical activity, providing a unique ‘natural experiment’ to understand the social factors associated with sustained physical activity. The objectives of this study were to identify the proportion of people who successfully sustained physical activity during lockdown and to explore whether social support, loneliness and social isolation were associated with maintenance of physical activity during COVID-19 lockdown.

**Method:**

Longitudinal data from 16,980 participants, mean age 51.3 years (SD = 14.3) from the COVID-19 Social Study was used to identify a sample of participants who maintained their physical activity despite lockdown.

**Results:**

Seventeen percent were consistently active whilst 42% were completely inactive. After adjustment for multiple confounders, high social support was associated with a 64% (95% CI 50–80%) increased odds of sustaining physical activity and medium social support was associated with 32% (95% CI 20–44%) increased odds. Associations between physical activity and loneliness and social isolation were not found.

**Conclusion:**

This study supports previous research showing the importance of social support for the long-term maintenance of physical activity behaviour but shows that such effects extend to contexts of social restrictions.

**Supplementary Information:**

The online version contains supplementary material available at 10.1007/s12529-022-10125-2.

## Introduction

In response to COVID-19 quarantine strategies such as lockdown, non-essential travel restrictions and social distancing were implemented in an attempt to reduce the spread of the virus [1]. The strategies are likely to have impacted the level and patterns of physical activity (PA) [2–4], with potential harmful effects on physical and mental health [1]. For example, in the United Kingdom (UK), gyms, leisure facilities and sports clubs were closed, affecting many usual exercise behaviours [5]. The pandemic led to major changes in commuter patterns, with many people working from home, furloughed or losing work, reducing active commuting [6]. Further, schools and childcare centres were closed, so home-based caring responsibilities increased for many, while decreasing the need to walk or travel [5]. Results from a systematic review of 66 articles looking at changes in PA during the COVID-19 pandemic showed the impact of such policies, with the majority of studies reporting a decrease in PA [7]. Despite restrictions, time outside to exercise was allowed, engaging in daily exercise was encouraged and meeting recommended daily activity levels was possible [5]. The pandemic restrictions therefore provide a ‘natural experiment’ to explore social determinants of PA behaviour. Identifying factors that are associated with successfully sustaining sufficient levels of activity despite significant barriers could help inform interventions and future pandemic responses. A retrospective observational study of 48,440 adults who were diagnosed with COVID-19 showed that people who consistently met the PA guidelines prior to the pandemic were associated with a reduced risk of severe COVID-19 outcomes (hospitalisation, admission to intensive care and death) [8], demonstrating the importance of maintaining PA during the pandemic.

To date, several studies have focused on *individual predictors* of decreases in PA during the COVID-19 pandemic. A UK smartphone-based tracking study (*n* = 5395) found a larger drop in PA during the first lockdown amongst younger people and those who had been active prior to lockdown [9], a finding echoed in a study of 532 Australian students [10]. However, other studies have found different results. A cross-sectional online study in Belgium (*n* = 13,515) reported that those aged < 55 years and were inactive prior to lockdown were likely to exercise more [11]. Of note, the mode of usual exercise appeared to be a key factor, with those who usually exercised with friends/sports clubs and who did not engage with online exercise tools reporting a reduction in exercise [11]. A study using the COVID-19 Social Study looking at trajectories of PA in relation to lockdown measures found that although 62% experienced little change, nearly 29% reduced PA and 12% of those who did not change were consistently inactive [12]. The majority of studies exploring predictors of changes in PA during the COVID-19 pandemic have been cross-sectional in nature and used a limited number of variables as predictors.

There is a lack of data to date exploring how individual social factors could have affected changes in PA during the pandemic. *Social support* (defined as the extent to which individuals perceive those around them are available to them and are attentive to their needs) has been associated with positive PA participation [13] and sustained PA prior to the COVID-19 pandemic [14]. Social support is not a single entity but multi-layered and complex with two main constructs: structural support and functional social support [15]. Structural support describes the existence of relationships and relates to the size, type and frequency of a social network. Functional support relates to the degree to which these relationships serve a function and provide resources [15] and incorporates instrumental (e.g. financial, practical help), emotional (e.g. empathy), informational (e.g. advice), companionship (e.g. sense of belonging) and validation (e.g. help builds one’s intrinsic value) [16]; all of these have been associated with PA before the pandemic [15, 17].

Stressful events may require multiple resources and types of support [18]. The effect of social support can be explained by two major hypotheses: the stress-buffering hypothesis, where it is thought social support can buffer the negative impact of stressful life events, and the direct-effect hypothesis, where social support has a positive effect on health, independent of stress levels [18]. People with high social support show overall better health in their daily lives [19].

There is also a distinction between actual support received in the past and perceived availability of support. Perceived social support refers to how individuals perceive friends, colleagues and family members as available to provide functional and overall support during times of need [20]. Perceived social support is regarded as a sensitive measure in the context of ability to cope with challenges and is related to better physical and mental health outcomes and quality of life [15]. Perceived social support has been found to have a significant positive effect on PA [21] and is used in the paper.

Social support may be particularly important during the pandemic as it has been shown to play a key role in PA participation and general well-being and is a strong predictor of resilience following disasters, e.g. Hurricane Katrina and exposure to trauma [22]. Specifically, social support may serve as a ‘buffer’ as per the stress-buffering hypothesis, providing emotional and psychological support, which is considered a major factor in maintaining well-being and coping with health challenges [23]. The importance of social support in relation to PA is well understood [13, 14, 24] with research indicating a positive relationship between social support, intention to be active and participation in physical activity [21, 25]; people with either general social support or PA-specific social support are more likely to participate in leisure time PA [14]. The greater the perceived social support, the less isolated and loneliness they experience, supporting increased intention and participation in PA.

The ongoing importance of social support has yet to be explored during the challenges of the pandemic. There is evidence that it might influence other health behaviours. For example, a cross-sectional study of changes in alcohol consumption in 1958 US university students (after COVID-19-related campus closure) showed those with greater perceived social support reported less alcohol consumption than those with lower social support [26].

Other social factors, including *social isolation* and *loneliness*, have also been related to PA pre-pandemic [27, 28], and levels may have increased as a result of lockdown restrictions. Social isolation and loneliness are distinct from, although related to, social support. Whilst social isolation refers to a lack of social contact with others, loneliness refers to the perception that one’s social contact is insufficient to meet one’s emotional needs [29, 30]. Social isolation has been shown to have a negative effect on the amount of overall physical activity, with an increase in social isolation directly related to reduced PA [28, 31]. Loneliness has also been identified as an independent risk factor for a reduction in activity and discontinuation of PA [32].

During the COVID-19 pandemic, social factors such as social isolation, loneliness and social support have all been affected. Quarantine and social distancing have led to elevated levels of loneliness and social isolation [33]. Cross-sectional results from the UK-based COVID-19 Psychological Wellbeing Study showed that rates of loneliness where high with a prevalence of 27% during the initial phase of lockdown [34], with the COVID-19 Social Study reporting a prevalence of 14% for severe loneliness [35]. Whether changes in individual-level experiences of social factors such as isolation, loneliness and social support have affected PA remains unknown.

In social epidemiology research, social isolation is the lack of meaningful social contacts, perceived isolation and having minimal people to interact with regularly [36]. In this paper, social isolation was conceptualised differently; it measures ‘isolation’ as defined by the UK Government during the first COVID-19 lockdown. The definition was ‘staying at home and avoiding contact with any people inside or outside the household’ [37]. This change from individual choice to isolation enacted by Government may have exposed different people to isolation.

Therefore, the aims of this study were to identify the proportion of people who successfully sustained PA during lockdown and to explore whether social support, loneliness and social isolation were associated with maintenance of PA during the first COVID-19 lockdown in the UK. We hypothesised that high social support would be favourably associated with PA, but loneliness and social isolation would have a negative impact on sustained activity. Our model is, therefore, that PA is a linear function of social support (positively), loneliness (negatively) and social isolation (negatively); see supplemental Fig. 1.

## Methods

### Study Design and Participants

Data was used from the COVID-19 Social Study (CSS), a large-scale, longitudinal, panel, observational study of adults (age ≥ 18 years) living in the UK during the COVID-19 pandemic [38]. The participants from the study are not randomly selected and therefore not representative of the UK population, but contains a heterogeneous sample [38]. Study participation required the following: aged ≥ 18 years, living in the UK, with a valid email address and internet access. Recruitment was undertaken using three primary approaches in order to make the study as representative as possible. Firstly, the study was promoted through the senior authors’ existing networks including large databases of adults who had previously consented to be involved in health research in the UK such as UCL BioResource, HealthWise Wales and through the UKRI Mental Health Research Networks. To ensure good heterogeneity and stratification over demographic groups, targeted recruitment was undertaken using advertising and recruitment companies focusing on (a) low-income backgrounds, (b) no or low qualifications, and (c) the unemployed. Finally, promotion via partnerships with third-sector organisations to vulnerable groups was undertaken. The CSS commenced on 21 March 2020. This study focused on the first 8 weeks of the pandemic during the period of full lockdown, when a single daily allowance of outdoor activity was allowed. Restrictions started to ease in England from 10 May 2020, when the allowance of exercise was changed to unlimited outdoor exercise. Changes in the restrictions followed in Wales and Scotland on 29 May 2020. A total of 69,475 people provided at least 1 week of data during the 8 weeks included in this study (see supplementary Table 1). Data was collected weekly via an online questionnaire. Baseline data was collected at wave 1 (wave 1 = the week participants joined the study); there were questions repeated weekly and one-off modules on a variety of topics. As data was collected online, completion of every question was required for submission. The study was approved by UCL Research Ethics Committee (12,467/005), with all participants giving informed consent.

Full documentation of data collection protocol is available at https://osf.io/jm8ra/

## Measures

### Dependent Variable

#### Physical Activity

Physical activity was self-reported on a weekly basis. Self-report questionnaires are the most common method of PA assessment: they are easy and accurate at measuring intense activity although less robust at measuring light to moderate activity [39]. A ‘*stylised questions*’ and ‘*time diaries*’ approach [40] was used to measure ‘time use’ of a specific set of activities including PA [41]. Participants were asked to focus on the last weekday, and report how much time they spent on three categories of PA. Although data collected prior to COVID-19 PA patterns suggested activity levels could be different between weekends and weekdays, with PA lowest on Sundays and highest on Saturdays in some studies [42], the average amount of time spent in moderate physical activity was not found to be significantly different between weekdays and weekends [43].

The three categories were gentle PA (e.g. walking slowly), moderate or vigorous physical activity (MVPA) (e.g. brisk walking, running, cycling, swimming) or exercise inside your home or garden (e.g. yoga, weights, indoor exercise). Time spent doing the different activities was reported as none, < 30 min, 30 min–2 h, 3–5 h and 6 + h.

The World Health Organization (WHO) defines physical activity as any bodily movement produced by skeletal muscles that requires energy expenditure, and current WHO and UK physical activity guidance recommends adults ≥ 18 years should aim to be active daily and achieve 150 min of moderate activity per week [44]. Benefits of PA are seen at even moderate levels of activity such as brisk walking and gardening for 30 min/day on most days of the week. Taking the description of moderate activity into account, the moderate/high intensity and in-home activity categories were combined to identify all those who would have achieved any kind of moderate activity levels. Those who reported < 30 min on the last weekday were felt unlikely to achieve the recommended 150 min/week of moderate activity and were designated as likely ‘inactive’; those who reported > 30 min were likely ‘active’. A description of long-term physical activity engagement was generated using a Physical Activity Pattern Index which consists of three ordered categories: inactive, intermittently active and active. Those who did not report active behaviour at any time point were classified as ‘inactive’; those categorised as ‘active’ 1–5 out of the 8 weeks were classified as ‘intermittently active’. Those categorised as ‘active’ 6–8 out of the 8 weeks (≥ 75%) were classified as ‘active’. Sustained physical activity is not a continuous behaviour; it is a process that may include episodes of sustained physical activity that can be discontinued for short or longer periods of time and resumed after setback, e.g. injury, illness [45–47].

### Independent Variables

#### Social Support

Although social support can be measured in a number of different ways, perceived social support is the most commonly measured index [48]. In this study, perceived social support was measured using the Perceived Social Support Questionnaire (F-SozU K-6) adapted for use in COVID-19 and reported weekly (see supplemental Table 2). This is a 6-item questionnaire with a 5-point Likert scale ranging from 1 = not at all to 5 = very true. The scores for each measure were then summed to give a total ranging from 6 to 30, where the higher the score, the higher the levels of social support. The sum score for social support was based on data at baseline (week 1). The questionnaire was reported in other studies to have excellent construct validity and reliability for perceived social support [49] with an internal consistency of 0.89 [50] and a Cronbach’s alpha of 0.86; they did not report on other relevant metrics such as face validity [51]. Recent research looking at the predictive role of social support amongst 325 frontline nurses in reducing COVID-19 anxiety [51] grouped people into three levels of perceived social support; scores of 6–17 = low, 18–25 = normal and 26–30 = high. Due to the skew towards normal/high social support, categorisation into three levels of support was easier to interpret than a continuous scale of support. Therefore, categorisation was adopted in this study.

#### Loneliness

Loneliness was measured using the UCLA-3 loneliness scale, a short form of the Revised UCLA Loneliness Scale (UCLA-R), and reported weekly. It is designed to measure subjective feelings of loneliness as well as feelings of social isolation; it is reliable and has strong validity [52]. This is a 3-item scale; respondents were asked how often they felt (1) they lack companionship, (2) left out and (3) isolated from others. Frequencies ranged from hardly ever (score = 1), some of the time (score = 2) and often (score = 3). The scores of each scale were summed to give a final score ranging from 3 to 9; the sum score for loneliness was based on data at baseline (week 1). A higher score of ≥ 6 indicates higher risk of loneliness. Researchers in the past have grouped people into the following categories [31]: scores of 3–5 = low risk of being lonely and scores of 6–9 = increased risk of being lonely. Categorisation was used due to how skewed the data was towards ‘not lonely’.

Social Isolation Status.

Due to the fast-moving nature of the lockdown and survey setup, the social isolation variable was only collected from week 4. Participants were asked about whether they were currently isolating in line with government guidelines. Only those who selected that they were in full isolation, not leaving their home and only interacting with their household for the full 5 weeks collected were categorised as socially isolated; therefore, social isolation was coded as a binary variable where 0 is not currently isolating and 1 is fully isolating in line with government COVID-19 guidelines.

### Covariates

We included data on various demographics: gender (male/female), age (18–29, 30–45, 46–59, 60 +), ethnicity (white vs BAME [black, Asian and minority ethnic]), a household income of > £30,000 p/a (yes/no), university education (degree or above vs high school or none) and employment (full-time, part-time employment or self-employed vs in education, unable to work, unemployed, homemaker or retired). Data were also collected on living alone (yes/no); urban living (living in a city or town vs living in a village or hamlet); physical health condition such as high blood pressure, diabetes or heart disease (yes/no); having a diagnosed mental health condition including depression, anxiety or any other mental health problem (yes/no); carer status (yes/no); key worker (those whose work is critical to the COVID-19 response including those in health and social care, education, key public services, local and national government, food and necessary good supply, public/national security, transport, utilities/communication and financial services) (yes/no); and active the week prior to lockdown (undertaking moderate to vigorous physical activity for ≥ 15 min on 5–7 days (to achieve 150 min of MVPA recommended by WHO and UK guidelines)). Variables were dichotomized where there were small numbers within the sub-variable or where there was no benefit in the level of data, e.g. differences in living in a village vs hamlet not relevant for PA, whereas rural vs urban is more useful; therefore, for the purpose of analysis, covariables were dichotomized where appropriate.

## Statistical Analysis

Analyses were carried out using Stata 14.0 (StataCorp, College Station, TX). Multiple imputation was used to account for missing data.

Ordered logistic regressions were performed in which PA was regressed individually onto all covariables: demographic (gender, age, ethnicity, income, education level and employment status), health (physical health condition, mental health condition, active prior to lockdown), living condition (lives alone, urban living) and other (carer or key worker). Ordered logistic regression was performed in which PA with loneliness, social support and social isolation were regressed on 3 models to identify if they influenced PA behaviour: model 1 adjusting for age and gender; model 2 additionally adjusting ethnicity, employment, income and education; and model 3 additionally adjusting for physical and mental health conditions.

## Results

Of the 27,271 participants who signed up to the CSS in week 1 of lockdown, 16,980 participants provided a minimum of 5 weeks of data (Table 1). Complete case analysis of the 8 weeks comprised 6906 participants and is provided in the online supplementary file. Our sample comprised 16,980 participants who started the study in week 1 and contributed at least 5 weeks of the 8 weeks included in this study; multiple imputation was performed for handling missing data.

**Table 1:** Baseline characteristics of participants (*n* = 16,980)

Variable	Total sample (*n* = 16,980)		Active (*n* = 2878)		Intermittent (6937)		Inactive (*n* = 7165)	
	***n***	***%***	***n***	***%***	***n***	***%***	***n***	***%***
**Gender**								
Female	12,653	*74.5*	849	*76.1*	2075	*75.5*	2192	*74.7*
Male	4250	*25.0*	262	*23.9*	659	*24.5*	837	*25.3*
**Age category**								
18–29	1238	*7.3*	272	*9.5*	611	*8.8*	355	*5.0*
30–45	4841	*28.5*	871	*30.3*	2203	*31.8*	1767	*24.7*
46–59	5469	*32.2*	917	*31.9*	2137	*30.8*	2415	*33.7*
60 +	5432	*32.0*	818	*28.4*	1986	*28.6*	2628	*36.7*
**Ethnicity**								
White	16,206	*95.5*	2698	*93.8*	6590	*95*	6918	*96.6*
BAME	774	*4.6*	180	*6.2*	347	*5.0*	247	*3.4*
**Income**								
> £30 K/year	11,137	*65.6*	4187	*58.4*	4814	*69.4*	2136	*74.2*
< £30 K/year	5843	*34.4*	2978	*41.6*	2123	*30.6*	742	*25.8*
**Education**								
≥ degree	11,813	*69.6*	2264	*78.7*	5098	*73.5*	4451	*62.1*
≤ high school	5167	*30.4*	614	*21.3*	1839	*26.5*	2714	*37.9*
**Employed**								
Yes	10,721	*63.1*	1944	*67.6*	4621	*66.6*	4156	*58.0*
**Key worker**								
Yes	3715	*21.9*	596	*20.7*	1584	*22.8*	1535	*21.4*
**Carer**								
Yes	2681	*15.8*	431	*15.0*	1062	*15.3*	1188	*16.6*
**Lives alone**								
Yes	3317	*19.5*	490	*17.0*	1214	*17.5*	1613	*22.5*
**Urban living**								
Yes	13,259	*78.1*	2329	*80.9*	5445	*78.5*	5485	*76.6*
**Chronic health condition**								
Yes	9042	*53.3*	1030	*35.8*	3062	*44.1*	3846	*53.7*
**Physical health condition**								
Yes	6844	*40.3*	858	*29.8*	2606	*37.6*	3380	*47.2*
**Mental health condition**								
Yes	2995	*17.6*	344	*12.0*	1103	*15.9*	1548	*21.6*
**Active prior to lockdown**								
Yes	4105	*24.2*	1286	*44.7*	1684	*24.3*	1135	*15.8*

### Missingness

A total of 27,271 participants signed up to the CSS in week 1 of lockdown. For the purpose of analysis, only those who entered the study at week 1 and had 5–8 weeks of data were included (*n* = 16,980) and full information on missingness per week for these participants is shown in Table 2. Logit regression was used to examine whether any of the variables included in the model predicted missingness; they did, and therefore, our assumptions were that data was ‘missing at random’. Multivariate imputation by chained equations was the method used to deal with missing data. The number of imputed datasets that was created was 5, with sex and age set as regular variables. Proportional odds assumption was tested using Brant test; assumptions hold for all independent variables.

**Table 2:** Missingness per week for participants who provided 5–8 weeks of data (total *n* = 16,980)

Week	Complete	Missing	% Missing
1	16,980	0	0
2	16,032	948	5.6
3	15,741	1239	7.3
4	15,718	1262	7.4
5	15,325	1655	9.8
6	14,510	2470	14.6
7	13,099	3881	22.9
8	13,389	3591	21.2

### Descriptive

Participant characteristics are presented in Table 1. Seventy-five percent were female, mean age was 51.3 years (SD = 14.3), 96% were white (British/Irish/other), 70% had degree level or above education, 63% were employed and 66% reported a higher income (above £30 k) threshold. Key workers accounted for 22% of the participants and 16% were carers. 53% reported a chronic long-term health condition, 40% stating a physical health condition and 18% a mental health condition, 24% reported being active the week prior to lockdown.

### Physical Activity

Reports of physical activity in an individual week ranged from 24.9% (week 1) at the lowest to 29.4% (week 4) at the highest. The Physical Activity Pattern Index, see Table 3, shows that 42% of participants were inactive, 41% were intermittently active and 17% were consistently active across the 8 weeks. Within the intermittently active group, the majority (59%) were active for only 1 or 2 weeks during the 8 weeks of the study. Fewest of the intermittent group (12%) were active for 5 weeks. Within the active group, there was a fairly even split of those active for 6, 7 or 8 weeks. Table 3 provides full details of the Physical Activity Pattern Index and within category results. A positive association with persistent PA behaviour, with no adjustment for covariates, was found with social support, being female, being BAME, having higher income, being employed, having a university-level education, urban living and being active prior to lockdown. Factors that were adversely associated with PA included loneliness, social isolation, living alone, having a physical or mental health condition and being aged 30 + (Table 4).

**Table 3:** Number of active weeks and Physical Activity Index

Number of active weeks	Number of participants	Total group %	Within category %
**Inactive *n* = 7165 (42%)**			
0	7165	42.2%	100%
**Intermittently active *n* = 6937 (41%)**			
1	2519	14.8%	36%
2	1510	8.9%	23%
3	1065	6.3%	14%
4	992	5.8%	15%
5	851	5.0%	12%
**Consistently active *n* = 2878 (17%)**			
6	893	5.3%	31%
7	1006	5.9%	35%
8	979	5.8%	34%
**Total**	**16,980**	**100**

**Table 4:** Effects of covariables on sustained physical activity (*n* = 16,980)

Physical activity	
**Variable**	Odds ratio (95% CI)
**Social support**	
Low	Reference
Medium	1.56 (1.44–1.69)***
High	2.05 (1.89–2.22)***
**Loneliness**	
Low	Reference
High	0.80 (0.75–0.85)***
**Social isolation (> wk 4)**	
Yes	0.66 (0.57–0.78)***
**Gender**	
Male	Reference
Female	1.11 (1.04–1.19)**
**Age**	
18–29	Reference
30–45	0.70 (0.61–0.80)***
46–59	0.51 (0.44–0.58)***
60 +	0.42 (0.38–0.49)***
**Ethnicity**	
White	Reference
BAME	1.59 (1.36–1.85)***
**High income**	
Yes	1.72 (1.62–1.84)***
**Employed**	
Yes	1.46 (1.37–1.56)***
**University education**	
Yes	1.83 (1.71–1.96)***
**Lives alone**	
Yes	0.82 (0.70–0.96)***
**Urban environment**	
Yes	1.17 (1.08–1.26)***
**Physical health condition**	
Yes	0.61 (0.57–0.65)***
**Mental health condition**	
Yes	0.63 (0.58–0.68)***
**Carer**	
Yes	0.90 (0.83–0.98)
**Key worker**	
Yes	1.04 (0.97–1.12)
**Active prior to lockdown**	
Yes	2.3 (2.14–2.49)***

*p < 0.05; **p < 0.01; ***p < 0.001.

### Social Support

There was an association between PA and social support. The mean social support score was ‘normal’ and ranged from 22.59 (SE 0.07) in week 1, with a slight decrease to 22 (SE 0.09) in week 8. Of those who were active, 13% had low social support, 41% had medium support and 46% had high support. Of the intermittently active, 15% had low social support, 43% had medium support and 42% had high support. Of the inactive, 23% had low support, 43% had medium support and 33% had high support. Ordered logistic regression demonstrated an increase in likelihood of being active amongst individuals with both medium and high support compared to those with low support (Table 4). High and medium social support continued to be positively associated with PA even when accounting for all demographic, health-related factors and other covariates (Table 5). High social support OR 1.64 (95% CI 1.5–1.8) *p* ≤ 0.001. Medium social support OR 1.32 (95% CI 1.2–1.44) *p* ≤ 0.001 (Table 5).

**Table 5:** Ordered logistic regression model of physical activity category (inactive, intermittently, active) with social support, loneliness and social isolation (*n* = 16,980)

Physical activity category	
**Variable**	**Odds ratio (95% CI)**
**Model 1—sex, age**	
Social support	
Medium	1.46 (1.34–1.59)***
High	1.89 (1.72–2.10)***
Loneliness	
Yes	0.92 (0.86–0.98)*
Social isolation	
Yes	0.77 (0.66–0.91)**
**Model 2—sex, age, ethnicity, employment status, income and education**	
Social support	
Medium	1.35 (1.24–1.47)***
High	1.7 (1.55–1.86)***
Loneliness	
Yes	0.95 (0.89–1.02)
Social isolation	
Yes	0.87 (0.74–1.02)
**Model 3—sex, age, ethnicity, employment status, income, education, chronic physical and mental health conditions**	
Social support	
Medium	1.32 (1.20–1.44)***
High	1.64 (1.50–1.80)***
Loneliness	
Yes	1.00 (0.96–1.10)
Social isolation	
Yes	0.97 (0.82–1.14)

*p < 0.05; **p < 0.01; ***p < 0.001.

### Loneliness

The mean loneliness score was ‘not lonely’ and ranged from 4.67 (SE 0.021) in week 1, with a slight increase in loneliness (shown by an increase in mean score) to 4.8 (SE 0.023) in week 8. The percentage of people who reported as lonely, per the UCLA scale, was 31.5% in week 1, increasing to 35.5% in week 8; a chi squared test showed no statistical difference in loneliness between week 1 and week 8.

There was a reduction in likelihood of being active amongst people who were lonely when accounting for sex and age, OR 0.92 (95% CI 0.86–0.98) *p* = 0.011 although the association was attenuated after further covariate adjustments (Table 5).

### Social Isolation

In unadjusted models, there was an association between isolation and lower odds of regular PA, OR 0.66 (95% CI 0.57–0.78) *p* ≤ 0.001. This was attenuated in further models (Table 5).

### Full Case Analysis

A total of 6906 participants provided data for all 8 weeks of the study. Analysis was replicated for a full case analysis, and findings were the same. Forty-four percent were classified as inactive, 40% as intermittently and 16% were active (supplemental Table 3) Ordered logistic regression showed high and medium social support continued to be positively associated with PA when compared with low social support even when accounting for all demographic, health-related factors and other covariates. High social support OR 1.74 (95% CI 1.49–2.02) *p* ≤ 0.001. Medium social support OR 1.29 (95% CI 1.13–1.49) *p* ≤ 0.001 (supplemental Table 4). Both loneliness and social isolation had a negative effect on PA; this association was attenuated in minimally adjusted models (supplemental Table 4).

## Discussion

The management of COVID-19 has created barriers for how people interact and maintain PA. In this large UK-wide study of adults, we identified a sub-sample of participants that were able to maintain their PA during lockdown despite restrictions. Those with high social support had a 64% increased odds; those with medium social support had 32% increased odds of sustaining PA during lockdown. However, associations between loneliness and social isolation had decreased odds of sustaining PA during lockdown. This was observed in minimally adjusted models, and the association was lost after adjusting for wider covariates.

When looking cross-sectionally at the data, levels of self-reported physical activity in our study are similar to those from other UK sources. For example, Sport England (2020) reported that 32% of adults were meeting the guidelines of 150 min/week MVPA in the last week of April 2020 (study week 6), whilst our study reported 27% active for the same week. Whilst both used self-reported PA, the Sport England participants are randomly selected households and data is weighted to the Office of National Statistics Populations measures, and therefore not directly comparable. However, our study highlights the difference between cross-sectional results and those who are meeting PA guidelines regularly. There is a risk that it could be less than the 32% reported by Sport England.

Such levels are concerning as they are lower than the estimated 63–66% of adults who met physical activity guidelines prior to COVID-19 [53]. However, our study built on previously reported cross-sectional data by showing that just 17% of adults analysed maintained recommended levels of physical activity throughout lockdown, 42% were inactive and a further 23% were active for only 1 or 2 weeks of the 8 weeks studied. This demonstrates the difference in those meeting the guidance when looking cross-sectionally compared to longitudinally and suggests that the number of people who were consistently active during the first UK lockdown could have been substantially lower than the cross-sectional reports. It is well known that not achieving the recommended levels of PA is associated with poor physical health, poor mental health and premature mortality [1]. This finding alone suggests that more work needs to be done on supporting peop during COVID-19 and potential future pandemics to meet PA guidelines on a regular basis in order to get maximum benefit from the activity.

Our study also explored what predicted the likelihood of an individual engaging in sustained PA across lockdown. Being white, well educated and a high earner; urban living; and good health status are all well-known predictors of PA [54, 55]. Any form of health condition, physical or mental health and older age are associated with a lower likelihood of being active [55]. Our findings were broadly in line with these pre-COVID-19 predictors [53].

We focused specifically on social predictors of sustained PA. Social support was found to be a consistent predictor, but loneliness and isolation were only associated in less-adjusted statistical models. The reasons for this may have been both direct and indirect. Directly, theories that are commonly used in PA interventions, e.g. Social Cognitive Theory, Theory of Planned Behaviour, Socio Ecological Model and Health Belief, all contain social support as a key factor in affecting behaviours [14]. The findings reported here suggest that even during social restrictions when such support may be disrupted from usual patterns (e.g. offered virtually rather than face to face), social support remains a key influencer of PA behaviours. Indirectly, it is also possible that social support may have played a role in buffering against the negative effects of poor mental health on PA during the pandemic. There is a large literature showing how mental health was adversely affected during the first UK lockdown [56]. Poor mental health is associated with lower PA engagement [57]. But research during the pandemic suggested that social interactions helped to reduce the experience of depressive symptoms, supporting the findings presented here [58]. Whilst the pandemic may have led to rises in loneliness and social isolation, this was situational due to lockdown. Chronic or prolonged social isolation and loneliness have a known negative impact on health and wellbeing [34], but it is possible that short-term loneliness and isolation do not have the same effect. Should there be multiple lockdowns, there is potential for the increased rates of loneliness to become chronic leading to it having an impact on physical activity.

The strengths of the study include its longitudinal design. It allows for multiple data points and for us to identify those participants who maintained their PA throughout lockdown. The sample provided information on a range of demographic factors, health conditions and social factors in addition to physical activity behaviours which has given us a unique opportunity to look at social isolation along with social support and loneliness. Limitations of the study include non-random sampling approach leading to a less representative sample of the UK population. As with many studies, participants were highly educated, white and female. The study used self-reported measure of PA leaving it open to reporting bias, e.g. imprecise recall. Attempts were made to minimise this by providing examples of common types of exercise with corresponding intensities. Asking participants to self-report on a single day of activity has its limitations; PA was one of thirteen measures of time use/activities which were collected. Due to concerns about focusing on a ‘typical’ day, which involves aggregating information from multiple days and averaging, a ‘time diary’ approach was used based on the previous weekday. There is potential that an ‘active’ person was allocated as ‘inactive’ if they had not undertaken physical activity on the previous working day. However, to achieve the WHO guidelines of 150 min MVPA/week, regular adherence should be ≥ 5 days/week of ≥ 30 min MVPA. To the best of our knowledge, the Covid Social Study (CSS) was the only study set up quickly enough to capture the first UK lockdown. With more time, additional variables and alternative validated questions may have been considered in the study providing better-quality PA data. This study looks at those who have remained active throughout lockdown; we are not aware of similar data published anywhere else looking at sustained activity.

The potential for multiple lockdowns over extended periods of time could cause prolonged periods of low PA for a substantial proportion of the population leading to increased risk of issues with physical and mental health. Previous research shows the importance of social support for initiating PA; this study demonstrates the importance of social support for the long-term maintenance of PA behaviour within the context of social restrictions and suggests that it does not need to be delivered face to face. Other social factors, such as loneliness and social isolation, were less consistent with their impact on PA. The development of interventions and programmes to support PA both during and outside of pandemic situations should ensure that social support is built in using theories that have shown to promote regular PA participation. The pandemic has prompted the development of virtual and remote PA through online classes and communities; supporting these programmes to build in social support could be beneficial to supporting regular PA both now and in the future.

VH was funded by the ESRC-BBSRC Soc-B Centre for Doctoral Training, ES/P000347/1. The Covid-19 Social Study was funded by Nuffield Foundation [WEL/FR-000022583], but the views expressed are those of the authors and not necessarily the Foundation’s. The study was also supported by the MARCH Mental Health Network funded by the Cross-Disciplinary Mental Health Network Plus initiative supported by UK Research and Innovation [ES/S002588/1], and by the Wellcome Trust [221400/Z/20/Z]. DF was funded by the Wellcome Trust [205407/Z/16/Z]. The researchers are grateful for the support of a number of organisations with their recruitment efforts including the UKRI Mental Health Networks, Find Out Now, UCL BioResource, SEO Works, FieldworkHub and Optimal Workshop. The study was also supported by HealthWise Wales, the Health and Care Research Wales initiative, which is led by Cardiff University in collaboration with SAIL, Swansea University. The funders had no final role in the study design; in the collection, analysis and interpretation of data; in the writing of the report; or in the decision to submit the paper for publication. All researchers listed as authors are independent from the funders, and all final decisions about the research were taken by the investigators and were unrestricted.

The research questions in the UCL COVID-19 Social Study built on patient and public involvement as part of the UKRI MARCH Mental Health Research Network, which focuses on social, cultural and community engagement and mental health. This highlighted priority research questions and measures for this study. Patients and the public were additionally involved in the recruitment of participants to the study and are actively involved in plans for the dissemination of findings from the study.

## Supplementary Information

Below is the link to the electronic supplementary material.

Supplementary file1 (DOCX 41 KB)
