# Supplementary material for: Perceived Social Support and Sustained Physical Activity During the COVID-19 Pandemic
Source: Int J Behav Med. 2022 Sep 29;30(5):651–62. doi: 10.1007/s12529-022-10125-2 (PMC9521870; doi:10.1007/s12529-022-10125-2)
Supplement: Supplementary file 1 — Supplementary file1 (DOCX 41 KB) [file 12529_2022_10125_MOESM1_ESM.docx]

**Online supplemental**

**Table 1.** Total number of participants providing data per calendar week.

| **Week commencing** | **Week** | **Frequency** |
| --- | --- | --- |
| 21/03/20 | 1 | 28,930 |
| 28/03/20 | 2 | 27,873 |
| 04/04/20 | 3 | 38,169 |
| 11/04/20 | 4 | 38,458 |
| 18/04/20 | 5 | 38,500 |
| 25/04/20 | 6 | 36,516 |
| 02/05/20 | 7 | 36,685 |
| 09/05/20 | 8 | 37,595 |

**Table 2.** Comparison of items in the original and revised Perceived Social Support Questionnaire (F-SozU K-6).

| Original | Adapted for COVID-19  In the past week, I feel… |
| --- | --- |
| I experience a lot of understanding and security from others | I have experienced a lot of understanding and support from others |
| I know a very close person whose help I can always count on | I have a very close person whose help I can always count on |
| If necessary, I can easily borrow something I might need from neighbours or friends | If necessary, I can easily borrow something I need from neighbours or friends |
| I know several people with whom I like to do things | I have people with whom I can spend time and do things together |
| When I am sick, I can without hesitation ask friends and family to take care of  important matters for me | If I get sick, I have friends and family who will take care of me |
| If I am down, I know to whom I can go without hesitation | If I am feeling down, I have people I can talk to without hesitation |

**Figure 1.** Model of relationship between social support, loneliness, social isolation and physical activity.


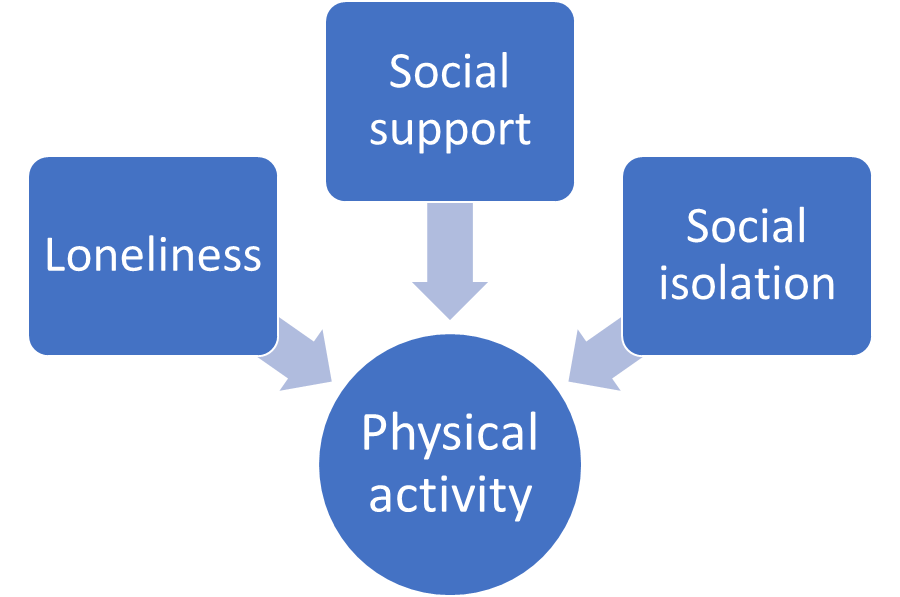


**Online supplemental – Full case analysis**

**Table 3.** Number of active weeks and Physical Activity Index (n=6906)

| **Number of active weeks** | **Number of participants** | **Total group %** | **Within category %** |
| --- | --- | --- | --- |
| **Inactive N=3043 (44%)** | | |  |
| 0 | 3043 | 44.1% | 100% |
| **Intermittently active N=2748 (40%)** | | |  |
| 1 | 964 | 14% | 36% |
| 2 | 629 | 9.1% | 23% |
| 3 | 398 | 5.8% | 14% |
| 4 | 436 | 6.3% | 15% |
| 5 | 321 | 4.6% | 12% |
| **Consistently active N=1115 (16%)** | | |  |
| 6 | 348 | 5% | 31% |
| 7 | 375 | 5.4% | 34% |
| 8 | 392 | 5.7% | 35% |
| **Total** | **6906** | **100** |  |

**Table 4.** Ordered logistic regression model of physical activity category (inactive, intermittently, active) with social support, loneliness and social isolation (n=6906)

| **Physical Activity Category** | |
| --- | --- |
| **Variable** | **Odds ratio (95% CI)** |
| **Model 1 – sex, age** |  |
| Social support  medium  high | 1.42 (1.25 – 1.62)***  1.95 (1.70 – 2.25)*** |
| Loneliness  Yes | 1.03 (0.93 – 1.15) |
| Social isolation  Yes | 0.79 (0.66 – 0.94)** |
| **Model 2 – sex, age, ethnicity, employment status, income and education** | |
| Social support  medium  high | 1.34 (1.17 – 1.54)***  1.81 (1.56 – 2.10)*** |
| Loneliness  Yes | 1.04 (0.92 – 1.16) |
| Social isolation  Yes | 0.88 (0.73 – 1.06) |
| **Model 3 - sex, age, ethnicity, employment status, income, education, chronic physical and mental health conditions** | |
| Social support  medium  high | 1.29 (1.13 – 1.49)***  1.74 (1.49 – 2.02)*** |
| Loneliness  Yes | 1.12 (1.00 – 1.26) |
| Social isolation  Yes | 1.00 (0.83 – 1.22) |

***P<0.05; **P<0.01; ***P<0.001**
